# Double-Sided Fabrication of Low-Leakage-Current Through-Silicon Vias (TSVs) with High-Step-Coverage Liner/Barrier Layers

**DOI:** 10.3390/mi16070750

**Published:** 2025-06-25

**Authors:** Baoyan Yang, Houjun Sun, Kaiqiang Zhu, Xinghua Wang

**Affiliations:** School of Integrated Circuits and Electronics, Beijing Institute of Technology, Beijing 100081, China; yangbaoyan@bit.edu.cn (B.Y.); sunhoujun@bit.edu.cn (H.S.)

**Keywords:** through-silicon via, parylene liner, double-sided process, electroless plating, electroplating

## Abstract

In this paper, a novel through-silicon via (TSV) fabrication strategy based on through-hole structures is proposed for low-cost and low-complexity manufacturing. Compared to conventional TSV fabrication processes, this method significantly simplifies the process flow by employing double-sided liner deposition, double-sided barrier layer/seed layer formation, and double-sided Cu electroplating. This method enhances the TSV stability by eliminating Cu contamination issues during chemical–mechanical polishing (CMP), which are a common challenge in traditional blind via fabrication processes. Additionally, the liner and barrier layer/seed layer achieve a high step coverage exceeding 80%, ensuring excellent conformality and structural integrity. For electroplating, a multi-stage bi-directional electroplating technique is introduced to enable void-free Cu filling in TSVs. The fabricated TSVs exhibit an ultra-low leakage current of 135 fA at 20 V, demonstrating their potential for advancing 3D integration technologies in heterogeneous integration.

## 1. Introduction

Three-dimensional integration technology has emerged as a key solution for sustaining Moore’s Law, offering improved circuit performance, power efficiency, and heterogeneous integration capabilities [1]. As a key component in 3D integration technology, through-silicon via (TSV) technology serves as a fundamental enabler of vertical interconnections in 3D integrated systems and has shown excellent capabilities in the heterogeneous integration and system-level packaging of multiple functional chips and devices, such as microelectromechanical systems (MEMSs), micro-sensors, integrated circuits (ICs), and radio frequency (RF) devices.

Typically, the fabrication of TSVs primarily relies on blind vias and normally involves the deposition of liners, barrier layers, seed layers, and conductors, thinning and chemical–mechanical polishing (CMP), the process of bonding support glass to protect the surface side, and the formation of redistribution layers (RDLs) [2,3,4]. It can be seen that the process flow associated with blind vias is complex, and polished Cu from the conductors used in the CMP process can potentially contaminate the substrate, posing reliability concerns. Thus extensive research efforts have focused on optimizing TSV technology, with a particular emphasis on the fabrication process, which is recognized as a critical yet challenging topic. In through-hole processes, both sides are coated with liners and seed layers; then the fabrication of the central conductor structures and redistribution layers is subsequently completed. Compared to blind via fabrication processes, through-hole processes eliminate the need for back-side liner deposition, barrier layer formation, and the associated photolithography processes. Thus, through-hole processes demonstrate advantages in reducing the fabrication complexity and costs, thereby avoiding CMP processes involving Cu pillars. Recent research has explored various double-sided TSV fabrication methods, including parylene-HT liners and serial sputtering techniques. However, these approaches often suffer from poor uniformity, a high cost, or complex process steps [5,6]. Additionally, polyimide has been adopted as a liner, where two sequential spin-coating steps enable double-sided deposition. This two-step process remains amenable to further simplification [7].

Considering the current research advancements, this work addresses these limitations through an innovative double-sided fabrication process. Conventional fabrication methods for double-sided TSV liners predominantly employ silicon dioxide as the dielectric material [8,9]. However, the fabricated double-sided SiO_2_ liners suffer from thermo-mechanical reliability issues due to the high Young’s modulus of SiO_2_ and significant coefficient of thermal expansion (CTE) mismatches among the liner, conductor, and substrate [10,11]. With the increasing adoption of polymer materials (e.g., parylene, polyimide) in MEMS fabrication owing to their superior electrical properties and reliability [12,13,14,15,16,17], numerous process schemes have been proposed for the fabrication of TSV and coaxial TSV structures utilizing polymer-based liners [18,19,20,21]. Nevertheless, achieving a double-sided architecture that combines simplicity, a low cost, and high step coverage remains technically challenging [22,23,24,25,26,27,28,29,30]. Traditional approaches for depositing barrier and seed layers on through-hole sidewalls primarily rely on multiple sputtering iterations [31,32,33]. These methods typically require four separate sputtering steps to sequentially process both the front and back sides of the through-hole structure. Furthermore, sputtered films often exhibit discontinuous coverage on via bottoms and a non-uniform thickness distribution across critical regions. Consequently, there remains an unmet requirement for a TSV fabrication methodology capable of simultaneously forming double-sided liners, seed layers, and central conductors through simplified process flows while delivering high reliability, superior step coverage, cost-effectiveness, and excellent electrical performance.

**Figure 1:**
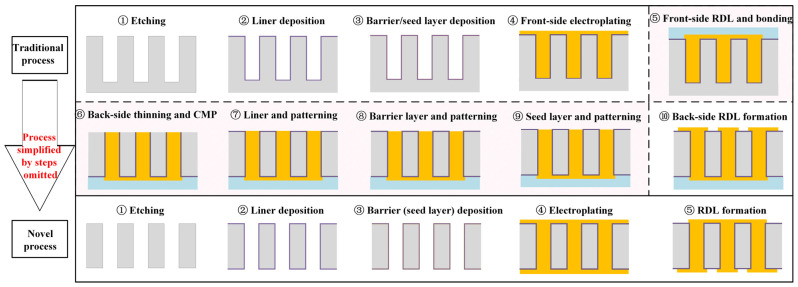
Comparison of the novel process and traditional process.

In this work, a novel TSV fabrication method based on through-hole structures is proposed (Figure 1). The method comprises five steps:

1. Etching through holes in the device wafer, which is attached to a support wafer by silicone oil with good thermal conductivity to enable etching;
2. The double-sided deposition of parylene liners via chemical vapor deposition (CVD);
3. Double-sided electroless nickel (Ni) plating to form barrier layers (which simultaneously serve as seed layers) in the TSV;
4. Double-sided electroplating for the complete filling of the structure;
5. Conducting front-side and back-side photolithography separately and RDL etching synchronously.

The parylene liner fabricated in this process exhibits a high step coverage and low relative dielectric constant. This property enhances the electrical isolation, reduces the signal loss, and ensures superior high-frequency performance. Ni, selected as the sidewall metallization material, acts as both a diffusion barrier layer and a seed layer [34]. The proposed electroless plating strategy enables the direct deposition of barrier layers/seed layers on both sides of the through-holes, thereby simplifying the metallization of deep vias by providing a bi-directional contact with the solution, demonstrating conformal layer formation. And finally complete filling is achieved through multi-stage double-sided electroplating. The new method simplifies the processes’ steps, reduces the processes’ execution times, and replaces some processes with lower-cost processes, thus significantly reducing the process costs and achieving the goal of low-cost preparation in TSV development.

## 2. Fabrication Flow and Results

### 2.1. Double-Sided Liner and Barrier Layer Deposition

Double-sided parylene-C deposition was implemented using a CVD system under optimized process conditions. Prior to deposition, a silicon wafer underwent sequential ultrasonic cleaning in acetone, isopropanol, and deionized water, followed by oxygen plasma activation to enhance the surface hydrophilicity. To improve adhesion, both the wafer surfaces were functionalized with a silane coupling agent through vapor-phase treatment.

**Figure 2:**
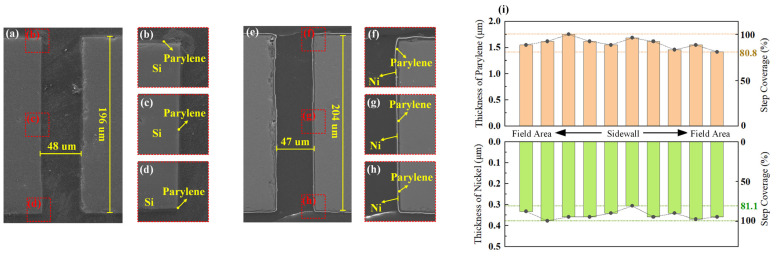
SEM images of the fabricated liner and seed layer and the thickness curves of these layers: (**a**) cross-sectional SEM images of the TSV after parylene liner deposition; (**b**) via top; (**c**) via middle; (**d**) via bottom; (**e**) cross-sectional SEM images of the TSV after seed layer deposition; (**f**) via top; (**g**) via middle; (**h**) via bottom; (**i**) step coverage results and comparison of the liner and seed layer.

The reaction parameters were precisely controlled, with dimer vaporization at 175 °C, pyrolysis at 690 °C, and polymerization at an ambient temperature. A 5.0 g parylene-C dimer precursor, encapsulated in an aluminum foil boat, was loaded into the vaporizer to initiate conformal growth. For simultaneous front/back-side coverage, the wafers were suspended using custom-designed minimal-contact fixtures (clamp area of <3% of the total surface), ensuring omni-directional monomer diffusion. The fabricated parylene liner exhibited >80% step coverage (Figure 2a–d).

Following parylene liner formation, electroless Ni plating was selected for the double-sided barrier layer/seed layer formation, utilizing Ni’s high density and effective Cu diffusion barrier properties. During the process development, the poor adhesion of palladium (Pd)-based catalyzators to parylene surfaces was discovered. To address this, a two-step surface activation was implemented by first using oxygen plasma treatment to roughen the parylene surface and then immersing it in 2.38% tetramethylammonium hydroxide (TMAH) at 25 °C under vacuum for 10 min to increase the number of hydrophilic groups on the material surface. Following oxygen plasma treatment and alkaline treatment, a greater number of hydrophilic functional groups were introduced to the parylene surface, along with a significant increase in the surface roughness of the thin film. These modifications enabled Pd ions, acting as catalysts, to provide more catalytic sites during the subsequent electroless plating process, thereby enhancing the adhesion strength of the deposited Ni thin film [35].

This method enabled conformal Ni deposition with a stronger adhesion effect, serving as both an effective barrier and a seed layer for subsequent Cu electroplating.

The process of double-sided electroless Ni plating on parylene liners was performed using conventional bath chemistry. The procedure consisted of four stages:

1. Surface cleaning with an alkaline solution;
2. Catalyst activation using a solution containing PdCl_2_/HCl;
3. Catalyst reduction in sodium hypophosphite;
4. Ni deposition using a Ni-P bath.

To ensure bi-directional uniformity, the silicon wafers underwent immersion inversion during processing, facilitated by a support structure that maintained full liquid contact while preventing air exposure.

Figure 2e–h also depicts the results of electroless Ni plating obtained using this method, showing the tightly adhered Ni seed layer on the parylene liner with demonstrable stability. The continuous Ni seed layer across the field areas, corners, and sidewalls of the TSV exhibited dense and uniform characteristics, with a thickness of approximately 360 nm and exceptional step coverage. The prepared Ni layer was consistent with the liner’s shape, demonstrating a uniform thickness and strong adhesion.

The thicknesses of the parylene and Ni layers resulting from these sequential processes were characterized using SEM, with measurements conducted of the field region, corners, and sidewalls of the structure. The data from these measurements are summarized in Figure 2i. As illustrated, the thickness of the parylene liner ranged from 1.4 to 1.75 µm, achieving a step coverage of 80%. Similarly, the thickness of the Ni seed layer varied from 300 to 360 nm, with a step coverage of 81%.

These results indicate that after seed layer preparation, the overall step coverage across the structure remained consistently above 80%, ensuring exceptional conformality. This consistency provided a robust foundation for subsequent processes.

### 2.2. Double-Sided Electroplating

**Figure 3:**
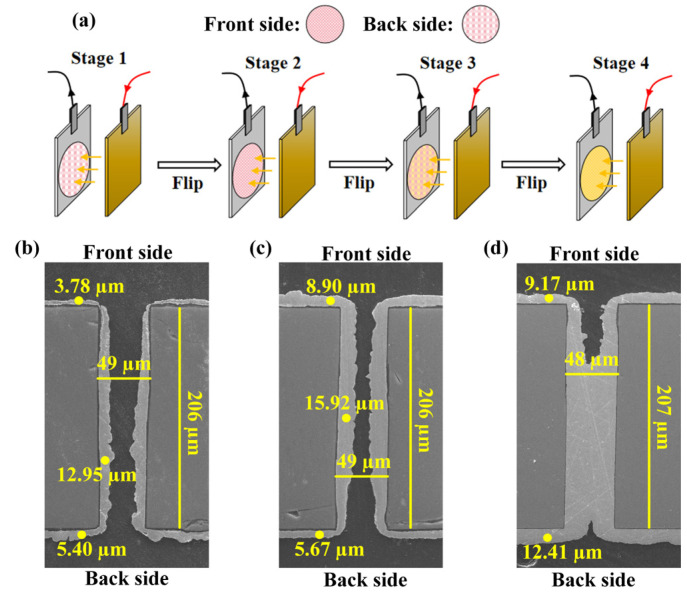
Schematic process flow of electroplating and SEM images of deposited Cu after different stages: (**a**) overall process flow; (**b**) SEM image after Stage 1; (**c**) SEM image after Stage 2; (**d**) SEM image after Stage 3.

When performing electroplating on through-silicon via (TSV) structures with identical aspect ratios, single-sided electroplating tends to induce more pronounced additive concentration gradients across the via compared to double-sided electroplating. This phenomenon arises from the inherently superior ionic transport efficiency in double-sided configurations, which facilitates the dynamic replenishment of additives within the via cavity during the deposition process. However, when targeting complete via filling, these approaches may encounter issues such as internal seam formation or an excessive Cu thickness in field regions. Therefore, a multi-stage single-sided electroplating technique was employed to achieve the complete filling of the TSV in this work, as shown in Figure 3a.

In Stage 1 (front-up electroplating), initial Cu deposition was performed in a front-up orientation, resulting in an electroplated metal layer on both the front side (3.78 µm) and back side (5.40 µm) of the silicon wafer. The via sidewall exhibited a maximum thickness of 12.95 µm, which was located in the mid–lower region of the TSV sidewall. To avoid the impact of an excessive Cu layer on subsequent processes (such as RDL preparation) and improve the electroplating uniformity, Stage 2 (front-down electroplating) was conducted by reversing the wafer orientation to a front-down position, enhancing back-side deposition. Stage 2 achieved front-side, back-side, and sidewall thicknesses of 8.90 µm, 5.67 µm, and 15.92 µm, respectively. This stage suppressed the local thickness accumulation usually present in single-sided processes, thereby partially restoring the electroplating uniformity.

Subsequent front-up electroplating in Stage 3 further increased the front-side and back-side thicknesses to 9.17 µm and 12.41 µm, respectively. The sealing of the lower–middle via region was achieved at this stage, initiating the use of a bi-directional bottom-up plating mechanism to eliminate central voids. Finally, in Stage 4 front-down deposition ensured complete via filling by balancing the interfacial growth rates, with no residual voids detected after the process. The material transport efficiency was better due to the through holes transporting additives from both the top and bottom directions, and the alternating front-up/front-down electroplating dynamically adjusted the electric field distribution and ionic flux direction, counteracting the inherent terminal effect in deep vias. The Stage 3 sealing of the lower–middle via region transitioned the deposition mode from surface-dominated to volume-dominated growth, ensuring progressive bottom-up filling. In the process, morphological refinement was first carried out through surface-dominated electroplating, followed by bottom-up thickening achieved by volume-dominated electroplating.

**Figure 4:**
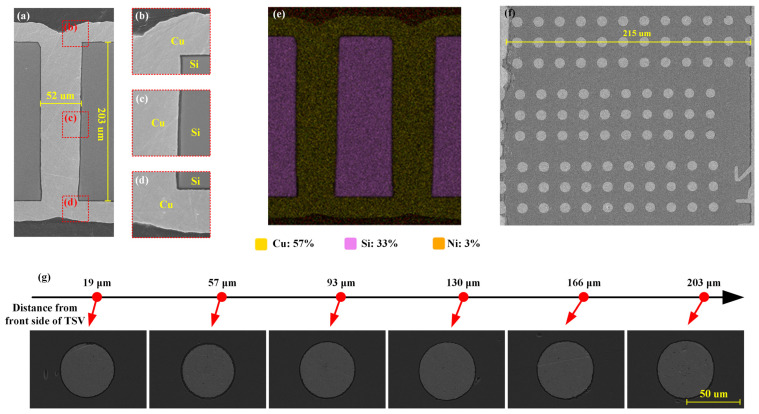
Fabrication results of TSV electroplating: (**a**) SEM image of overall TSV structure; (**b**) via top; (**c**) via middle; (**d**) via bottom; (**e**) EDS full-element scanning results; (**f**) SEM image of top-down inspection; (**g**) enlarged SEM image of top-down inspection results at different depths.

Figure 4 illustrates the central conductor formed using this method; the diameter and height of the TSV were 50 µm and 210 µm. SEM and EDS images confirmed the presence of dense Cu pillars within the TSVs. Top-down inspection revealed compact Cu deposition across all the via depths (Figure 4f,g), proving that the TSV was fully filled by the proposed electroplating process, ensuring optimal electrical performance and reliability.

Throughout the entire process, it is crucial to note that the liner and seed layer deposition step maintained the fundamental morphology of the through via, particularly assisting in achieving excellent step coverage. Initially, during the deposition of the double-sided parylene liner within the through via, an ultra-high step coverage was achieved across the field region, sidewalls, and corners of the liner. Subsequently, precise control over the electroless Ni plating process was implemented by optimizing the solution composition and adjusting the deposition temperature and airflow. During the conductive filling stage, front-up electroplating established conformal layers, followed by front-down deposition to balance the interfacial growth. Subsequent front-up plating was induced via sealing in the middle of the TSVs, transitioning to bi-directional bottom-up filling, and a final front-down electroplating step ensured complete via filling. The TSV structure obtained using this electroplating scheme had uniform Cu layers with a suitable thickness on both sides, providing the possibility for the subsequent direct double-sided preparation of RDL structures, eliminating the potential need for a CMP process, and simplifying the RDL preparation process. By integrating the fabrication of the double-sided liners and seed layers, eliminating the need for additional barrier layer preparation, and employing a double-sided electroplating strategy, the process effectively reduced the steps and costs while achieving the fabrication of reliable TSV structures.

## 3. Evaluation and Discussion

**Figure 5:**
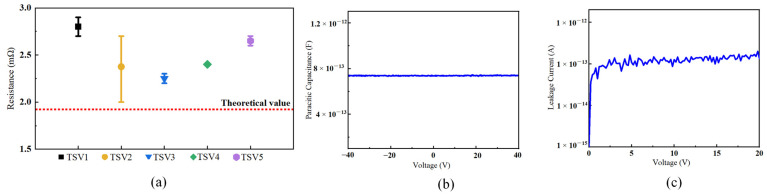
Measured electrical performance of fabricated TSVs: (**a**) resistance measurements of TSVs; (**b**) parasitic capacitance measurements of TSVs; (**c**) leakage measurements of TSVs.

The prepared TSV structures underwent electrical characterization using the Keysight B1500A semiconductor test analyzer, including measurements of the resistance, capacitance, and leakage current. For the resistance measurement, a Kelvin structure was employed. Each TSV structure was tested 400 times. Figure 5a presents the measured resistance values, ranging from 2 to 3 mΩ for each TSV structure, close to the theoretical value. The consistency between the measured TSV resistance and the theoretical value demonstrates the efficacy of the fabrication processes in achieving high-quality Cu filling with minimal voids or interfacial defects, as minor deviations are often attributed to residual stresses or minor geometric variations. This low resistance value also confirms the structural integrity of the TSVs, ensuring minor ohmic losses, which is critical for maintaining the power delivery efficiency and signal integrity in 3D ICs.

Capacitance–voltage (C-V) tests were conducted on individual TSV structures, as shown in Figure 5b, indicating a capacitance value of approximately 738 fF. The stable parasitic capacitance measured indicates effective dielectric isolation. This capacitance also confirms the uniformity of the parylene liner. This capacitance could reduce the dynamic power losses and crosstalk in high-speed interconnects, meeting the 3D IC requirements for TSVs.

**Figure 6:**
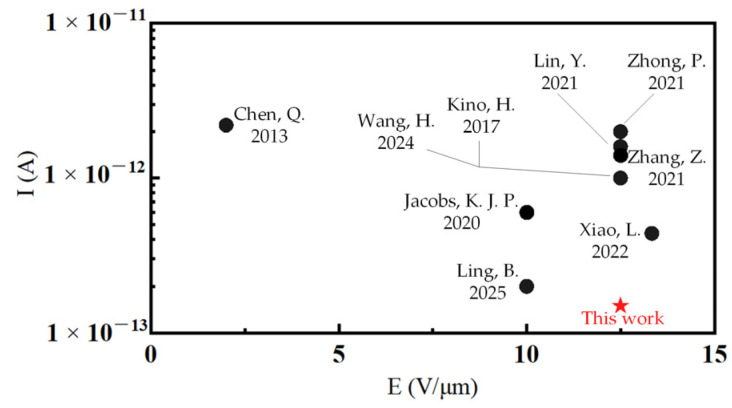
Comparison of the leakage current of the TSV structure in this work and others [22,36,37,38,39,40,41,42,43].

Figure 5c illustrates the test results for the leakage current between the Cu conductor and silicon substrate of a single TSV structure. The tests showed a leakage current of only 135 fA at a 20 V voltage, corresponding to a leakage current density of 0.390 nA/cm^2^. As demonstrated in Figure 6, the proposed TSV structure exhibited the lowest leakage current in structures with similar electric field intensities to those referred to in [22,36,37,38,39,40,41,42,43]. This low leakage current density underscores the excellent isolation performance of the liner structure and process. The advantages of a low leakage current in TSV technology include a minimized signal degradation risk, the stable operation of integrated circuits, reduced power dissipation, enhanced power management effectiveness, reduced electrical noise, clearer signal transmission, and improved overall signal integrity. These benefits potentially lower the manufacturing costs and improve the economic feasibility of advanced TSV technology.

**Figure 7:**
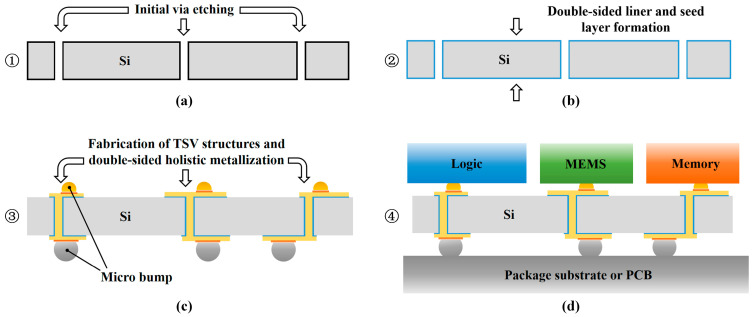
Schematic application of the interposer in TSV fabrication using the novel process: (**a**) initial via etching; (**b**) double-sided liner and seed layer formation; (**c**) fabrication of TSVs and micro bumps; (**d**) package in the device.

Figure 7 demonstrates the schematic of the interposers applied in the novel process. In the novel process, the interposer applies a double-sided deposition of the liner and seed layer after through via etching, subsequently employing double-sided electroplating with synchronized patterning to form double-sided RDL structures. Ultimately, solder joints are formed to create the interposer that is positioned directly between the packaging substrate and upper functional modules. Compared with conventional interposers fabricated through sequential blind via processing, the proposed double-sided process achieves enhanced process integration and improved structural consistency and reliability: it eliminates repeated wafer flipping and stepwise deposition, reducing the photolithography mask usage and shortening the production cycle. Simultaneous double-sided deposition mitigates the stress asymmetry caused by sequential processing and minimizes mechanical damage/misalignment during flipping, enhancing the electrical stability of TSV-RDL interconnects.

This approach is particularly suitable for high-density 2.5D/3D integration requiring high-density vertical interconnection and low parasitic parameters, offering a scalable manufacturing solution for next-generation heterogeneous integration. The double-sided synchronous process provides system-level benefits in advanced packaging applications. The symmetrical interconnect architecture reduces the signal path complexity and enhances the high-frequency signal integrity, addressing 5G millimeter-wave communications and AI accelerators’ demands for low latency and high bandwidth density. The structure also supports the heterogeneous integration of denser I/O arrangements while suppressing crosstalk. The low-residue seed layer etching minimizes the edge effects under high-frequency electromagnetic fields, offering a superior platform for silicon photonics integration.

## 4. Conclusions

In this work, a novel TSV fabrication method based on through-hole structures was proposed. The method provides low fabrication costs and simplified procedures by eliminating the use of multiple liners, barrier layer deposition, and the CMP and photolithography steps required in conventional approaches. According to the novel process scheme proposed in this paper, the liner and seed layer at all structural locations are directly fabricated immediately after via etching; simultaneously, this paper also developed a double-side electroplating process based on through-hole structures which achieves the fabrication of a final dense and fully filled structure through multi-stage electroplating without requiring additional processes or auxiliary layers. Moreover, the deposited liner and barrier layers demonstrate exceptional step coverage exceeding 80%. The electrical performance demonstrates a leakage current as low as 135 fA at 20 V. This work holds strong potential to play a significant role in the advancement of three-dimensional integration technology. In future work, the implementation and results of this new double-sided method in high-aspect-ratio TSV structures will be discussed, and research on the application of this method in TSV structures for high-frequency and other application scenarios, as well as performance testing studies, will also be conducted as part of future work.

## Data Availability

The data presented in this study are available on request from the corresponding authors. The data are not publicly available due to privacy.

## Abbreviations

The following abbreviations are used in this manuscript:

TSV	Through-silicon via
CMP	Chemical–mechanical polishing
MEMS	Microelectromechanical system
IC	Integrated circuit
RF	Radio frequency
RDL	Redistribution layer
CTE	Coefficient of thermal expansion
CVD	Chemical vapor deposition
